# Occupational injuries entail longer sick leave than non-occupational ones: evidence from a developing country and possible implications

**DOI:** 10.2478/aiht-2026-77-4165

**Published:** 2026-09-25

**Authors:** Nada Marić, Jovanka Koprena, Petar Bulat

**Affiliations:** University of Banja Luka Faculty of Medicine, Banja Luka, Bosnia and Herzegovina; Institute of Occupational Health and Sports Medicine, Banja Luka, Bosnia and Herzegovina; University of Belgrade Faculty of Medicine, Belgrade, Serbia; Serbian Institute of Occupational Health, Belgrade, Serbia

**Keywords:** absenteeism, non-work-related sick leave, temporary work incapacity, work-related sick leave, apsentizam, bolovanje zbog ozljede izvan rada, bolovanje zbog ozljede na radu, privremena nesposobnost za rad

## Abstract

Occupational injuries are an important public health and socioeconomic issue due to their effects on morbidity, productivity, and healthcare costs. The objective of this cross-sectional study was to compare the frequency of occupational and non-occupational injuries, the duration of respective sick leaves, and whether it is related to demographic and clinical factors. To this end, we analysed the data obtained from the information system of the Institute of Occupational and Sports Medicine of the Republic of Srpska, Bosnia and Herzegovina. The study included 2,075 patients with temporary work incapacity lasting longer than 30 days registered between 1 January and 31 December 2024. Variables included gender, age, ICD-10 diagnosis, and duration of sick leave. The economic burden of occupational injuries was estimated by calculating excess lost workdays compared to those lost to non-occupational injuries and multiplying them by the average gross daily wage in the Republic of Srpska. To approximate employer-related costs, including productivity loss and direct costs, were multiplied by a factor of two. Occupational injuries accounted for 46.2 % of cases and non-occupational injuries for 53.8 %. The mean duration of temporary work incapacity was significantly longer for occupational injuries (91.1±71.5 days) than for non-occupational injuries (78.4±49.6 days; p<0.001). Age significantly influenced sick leave duration, with the longest absences observed in workers aged ≥51 years. Occupational injuries resulted in 12,167 more lost workdays than non-occupational injuries, with an estimated total economic burden of about €885,000. Our findings show that, although less frequent, occupational injuries cause longer sickness absence and greater economic burden and therefore call for improved prevention and post-injury management strategies.

## Abstract (hr)

Ozljede na radu značajan su javnozdravstveni i socioekonomski problem zbog svojega utjecaja na morbiditet, produktivnost i troškove zdravstvene zaštite. Cilj ovog presječnog ispitivanja bio je usporediti učestalost ozljeda na radu i izvan rada, trajanje pripadajućih bolovanja te ispitati njihovu povezanost s demografskim i kliničkim čimbenicima. U tu svrhu analizirani su podatci iz informacijskoga sustava Zavoda za medicinu rada i sporta Republike Srpske u Bosni i Hercegovini. Ispitivanje je obuhvatilo 2.075 pacijenata s privremenom nesposobnošću za rad koja je trajala duže od 30 dana u razdoblju 1. siječnja – 31. prosinca 2024. Analizirane varijable uključivale su spol, dob, dijagnozu prema MKB-10 (ICD-10) i trajanje bolovanja. Ekonomsko opterećenje povezano s ozljedama na radu procijenjeno je izračunom viška izgubljenih radnih dana u odnosu na ozljede izvan rada i njihovim množenjem s prosječnom bruto dnevnom zaradom u Republici Srpskoj. Radi približne procjene ukupnih troškova za poslodavca, uključujući gubitak produktivnosti i izravne troškove, dobiveni iznos pomnožen je faktorom dva. Ozljede na radu činile su 46,2 % svih slučajeva, a one izvan rada 53,8 %. Prosječno trajanje privremene nesposobnosti za rad zbog ozljede na radu bilo je značajno duže (91,1±71,5 dana) nego zbog ozljeda izvan rada (78,4±49,6 dana; p<0,001). Dob pacijenata značajno je utjecala na trajanje bolovanja: najduže odsutnosti s posla zabilježene su kod radnika koji su navršili 51 godinu i starijih. Zbog ozljeda na radu izgubljeno je čak 12.167 radnih dana više u usporedbi s ozljedama izvan rada, uz procijenjen ukupan ekonomski trošak od približno 885 tisuća eura. Rezultati ispitivanja pokazuju da ozljede na radu, premda su manje učestale, dovode do dužeg bolovanja i većeg ekonomskog opterećenja, zbog čega je potrebno poboljšati preventivne mjere i strategije oporavka radnika.

Despite continuous improvements in occupational health and safety standards, about two million people die from occupational injuries and occupational diseases each year worldwide, placing them among the leading global public health and socioeconomic concerns, especially in low and middle-income countries (1, 2).

Besides impaired health and temporary or permanent loss of work ability, occupational injuries incur medical treatment and rehabilitation costs as well as substantial indirect costs of impaired or disrupted productivity. For example, a study examining the economic burden of occupational injuries and diseases in five European Union countries (3) reported that these costs ranged from 2.7 % to 10.4 % of the gross domestic product (GDP). Additionally, these consequences extend to the family and social life of workers, highlighting the multidimensional nature of occupational trauma (2).

Current literature (4,5,6,7,8) increasingly emphasises that the burden of occupational injuries should not be assessed solely in terms of incidence but also through their outcomes, particularly with regard to the duration of temporary work incapacity.

In the Republic of Srpska, a confederate unit of Bosnia and Herzegovina, an increasing trend has been observed in the number of mild to fatal occupational injuries between 2020 and 2024, while sectoral distribution highlights a pronounced concentration of risk in industries such as construction, transport, forestry, and healthcare (9). Similar patterns are evident at the level of the European Union (10), where certain sectors consistently bear the highest burden of occupational injuries. However, the Republic of Srpska does not follow the methodology used for reporting the European Statistics on Accidents at Work, as it defines fatal occupational injuries exclusively as those resulting in death at the moment of injury, and its definition of occupational injuries also includes injuries sustained during commuting to and from work (11).

Occupational and non-occupational injuries differ not only in their circumstances of occurrence but also in their legal, administrative, and compensation frameworks. To our knowledge, similar studies comparing these two groups in terms of the frequency and duration of sickness absence have not been conducted in the Republic of Srpska, yet may provide valuable information and help identify opportunities to improve occupational health policies, rehabilitation strategies, and management of sickness absence. The aim of this study was therefore to compare the frequency and characteristics of occupational and non-occupational injuries in a developing economy such as that of the Republic of Srpska, Bosnia and Herzegovina.

## METHODS

This cross-sectional study included the anonymous public records of 2,075 patients, all of whom were granted sick leave for more than 30 days due to occupational or non-occupational injuries by the First-Instance Medical Commission for the Assessment of Temporary Work Incapacity which started and ended between 1 January and 31 December 2024. Cases that did not start and end within this study period were excluded as incomplete to measure the total duration of sickness absence. Similar to other European countries, the First-Instance Commission for the Assessment of Temporary Work Incapacity is a multidisciplinary expert panel appointed by the state-owned universal health insurance fund to assess and grant or reject claims for continued sick leave based on medical documentation (12, 13).

The study was conducted in accordance with the principles of the Declaration of Helsinki. The analysis was based on routinely collected and processed anonymous medical data obtained from the information system of the Institute of Occupational and Sports Medicine of the Republic of Srpska. No direct contact was made with patients during the study.

The following data were collected: gender, age, sick leave start and end date in 2024, and diagnosis according to the International Classification of Diseases, 10^th^ Revision (ICD-10) (14), as this classification was used officially during the study period, even though ICD-11 became available by that time.

To estimate the potential economic burden associated with occupational injuries, we first calculated the number of lost workdays by multiplying the number of recorded occupational injury cases by the mean duration of sick leave due to occupational injuries. The same method was used to calculate the number of lost workdays due to non-occupational injury. To account for different sample size between occupational and non-occupational injury cases, we did not opt for direct comparison of lost workdays but opted for a standardised approach using the mean duration of sick leave as a reference value. Then we calculated the difference between these two mean values and multiplied it by the average gross daily wage in the Republic of Srpska, calculated from the average gross monthly salary. Furthermore, to approximate the broader economic impact on employers, including both salary compensation and productivity loss or replacement workforce costs, the obtained burden was multiplied by a factor of two.

### Statistical analysis

Patients were divided in two groups: those with occupational injuries and those with non-occupational injuries. For numerical variables we calculated means and standard deviations (SD), while categorical variables are presented as frequencies and percentages (%). To compare large categorical datasets between the two groups we used the chi-squared test of independence. To compare small categorical datasets, we used Fisher's exact test.

Normality of distribution was assessed with the Kolmogorov-Smirnov and Shapiro-Wilk test. To compare normally distributed numerical variables between two independent groups we used the independent samples *t*-test, and for those without normal distribution we used the Mann-Whitney *U* test.

For comparisons involving more than two independent groups, we used one-way analysis of variance (ANOVA) for normally distributed data and the Kruskal-Wallis test for non-normally distributed data, followed by rank-sum tests for post hoc comparisons (Mann-Whitney *U* test with Bonferroni correction).

Statistical significance was set to p<0.05. All statistical analyses were run on the SPSS for Windows version 25 software package (IBM, Armonk, NY, USA).

## RESULTS

Table 1 shows the demographic and injury data of the study population. The mean patient age was 45.04±12.2 years (range: 19–65), and the largest percentage belonged to the oldest age group (38.35 %).

**Table 1:** Frequency of occupational and non-occupational injuries in the study population, stratified by gender, age, and diagnosis

	**Total injuries N (%)**	**Occupational injuries N (%)**	**Non-occupational injuries N (%)**	**p**
**Gender**				
Male	1261 (60.8)	574 (45.5)	687 (54.5)	0.47
Female	814 (39.2)	384 (47.2)	430 (52.8)	
**Age (years)**				
≤30	318 (16.2)	132 (41.5)	186 (58.5)	0.13
31–40	373 (19.0)	167 (44.8)	206 (55.2)	
41–50	523 (26.6)	250 (47.8)	273 (52.2)	
≥51	750 (38.2)	366 (48.8)	384 (51.2)	
**Category of injury**				
Occupational injury	958 (46.2)	/	/	
Non-occupational injury	1117 (53.8)	/	/	
**ICD-10 diagnosis**				
S00–S19: Head and neck injuries	261 (12.6)	161 (61.7)	100 (38.3)	<0.001
S20–S39: Thorax, abdomen, lower back, pelvis injuries	241 (11.6)	123 (51.0)	118 (49.06)	
S40–S69: Upper extremity injuries	666 (32.1)	322 (48.3)	344 (51.7)	
S70–S99: Lower extremity injuries	854 (41.2)	320 (37.5)	534 (62.5)	
T00–T99: Multiple injuries, poisoning and other consequences of external causes	32 (1.5)	18 (56.3)	14 (43.8)	
V01–Y98: External causes of morbidity and mortality	21 (1.0)	14 (66.7)	7 (33.3)	

Non-occupational injuries in our population prevailed over the occupational ones. The most common were injuries to the lower extremities, followed by injuries to the upper extremities. The distribution of injuries was balanced between the genders.

Table 2 shows mean durations of temporary work incapacity (sick leave) and their distribution by gender, age, occupational v non-occupational category, and ICD-10 diagnosis. Mean sick leave was significantly longer in occupationally injured patients than those with non-occupational injuries (p<0.001). Furthermore, it was significantly longer in occupationally injured men than women (p=0.03). The duration of temporary work incapacity rose significantly with age, which is also true for occupational injuries. As regards ICD-10 diagnoses, patients with occupational thoracal, abdominal, lower back, pelvic, and lower extremity injuries had significantly longer sick leaves than patients with the same non-occupational diagnosis.

**Table 2:** Duration of sick leave due to injury in the study population, stratified by gender, age group, category of injury (occupational or non-occupational), and diagnosis

		**N**	**Mean**	**SD**	**Min**	**Max**	**p**
**Total**		2075	85.69	61.22	31	365	
**Category of injury**							
Occupational injury		958	91.16	71.54	31	365	**<0.001**
Non-occupational injury		1117	78.43	49.62	31	365	
**Category of injury**	**Gender**						
Occupational injury	Male	574	98.83	75.83	31	365	**0.03**
	Female	384	87.19	64.06	31	365	
Non-occupational injury	Male	687	79.44	50.85	31	365	0.17
	Female	430	76.81	47.62	31	365	
**Gender**	**Category of injury**						
Male	Occupational injury	574	98.83	75.83	31	365	**<0.001**
	Non-occupational injury	687	79.44	50.85	31	365	
Female	Occupational injury	384	87.19	64.01	31	365	**<0.001**
	Non-occupational injury	430	76.81	47.62	31	365	
**Age (years)**							
≤30		318	76.23	51.35	31	365	**<0.001**
31–40		373	78.28	57.38	31	365	
41–50		523	86.29	57.37	31	365	
≥51		750	87.65	68.19	31	365	
**Category of injury**	**Age (years)**						
Occupational injury	≤30	132	85.44	66.22	31	365	**0.01**
	31–40	167	85.38	66.04	31	365	
	41–50	250	93.96	68.42	31	365	
	≥51	366	92.97	76.49	31	365	
Non-occupational injury	≤30	186	69.70	36.18	31	311	**0.01**
	31–40	206	72.53	48.68	31	365	
	41–50	273	79.27	43.89	31	301	
	≥51	384	84.64	58.21	31	365	
**Age (years)**	**Category of injury**						
≤30	Occupational injury	43	85.44	66.22	31	365	**<0.001**
	Non-occupational injury	68	69.70	36.18	31	311	
31–40	Occupational injury	132	85.38	66.04	31	365	<0.001
	Non-occupational injury	186	72.53	48.68	31	365	
41–50	Occupational injury	167	93.96	68.42	3	365	<0.001
	Non-occupational injury	206	79.27	43.89	31	301	
≥51	Occupational injury	250	100.83	76.49	31	365	<0.001
	Non-occupational injury	273	84.64	58.21	31	365	
**ICD-10 diagnosis**							
S00–S19: Head and neck injuries		261	65.40	37.53	31	301	**<0.001**
S20–S39: Thorax, abdomen, lower back, pelvis injuries		241	93.93	74.07	31	365	
S40–S69: Upper extremity injuries		666	81.80	52.34	31	365	
S70–S99: Lower extremity injuries		854	90.65	63.61	31	365	
T00–T99: Multiple injuries, poisoning and other consequences of external causes		32	130.90	112.13	31	365	
V01–Y98: External causes of morbidity and mortality		21	85.67	96.23	32	365	
**ICD-10 diagnosis**	**Category of injury**						
S00–S19	Occupational injury	161	63.69	31.76	31	202	0.99
	Non-occupational injury	100	68.16	45.36	31	301	
S20–S39	Occupational injury	123	103.76	82.54	31	365	**0.05**
	Non-occupational injury	118	83.69	62.81	31	365	
S40–S69	Occupational injury	322	88.07	62.02	31	365	0.06
	Non-occupational injury	344	75.94	40.51	31	365	
S70–S99	Occupational injury	320	108.39	79.14	31	365	**<0.001**
	Non-occupational injury	534	80.01	49.22	31	365	
T00–T99	Occupational injury	18	148.11	111.99	49	365	0.24
	Non-occupational injury	14	124.79	115.22	31	365	
V01–Y98	Occupational injury	14	106.07	113.46	32	365	0.21
	Non-occupational injury	7	44.86	7.76	35	59	

The analysis included 958 occupational injuries, which resulted in a total of 87,274 sick leave days. The mean duration of sick leave per occupational injury was 91.1 days. For comparison, if the same number of cases had followed the average duration of absence for non-occupational injuries (78.4 days), the expected total number of sick leave days would have been 75,107 days. The difference between the observed and expected values indicates an excess of 12,167 sick leave days attributable to occupational injuries.

From this excess and average daily gross wage equivalent to €36, the estimated direct additional cost of sickness absence was around €440,000, while the indirect costs, which includes workforce replacement and productivity loss was twice as high, i.e. around €880,000.

## DISCUSSION

Our findings indicate that occupational injuries, although less frequent than non-occupational in the study sample, involve significantly higher mean duration of sick leave than non-occupational injuries. This suggests greater severity or complexity of the injuries sustained at the workplace but also reflects the influence of administrative, organisational and legal factors, including compensation systems. In the Republic of Srpska, the compensation for temporary work incapacity due to occupational injuries amounts to 100 % of the salary for the entire period of sick leave. It is covered by the employer. In contrast, the compensation for non-occupational injuries is typically 70 % and is covered by the government-owned Health Insurance Fund (13, 15). In both cases, the assessment of temporary work incapacity and approval of the sick leave are conducted by a commission appointed by this Health Insurance Fund.

Our results are in line with a Spanish study (4) reporting longer durations of sick leave following occupational injuries, despite their lower overall frequency, and we find them an important contribution to the limited current evidence, highlighting the need for further research to pinpoint the reasons for this phenomenon. For now, we can only speculate that one of the most influential factors may be the entitlement to full sick leave compensation, as partial compensation may force non-occupationally injured patients to return to work earlier, even though this may increase the risk of incomplete recovery, recurrent absence, or long-term adverse health outcomes.

Regarding gender differences, we found no significant difference in injury frequency between men and women, even though some studies (16,17,18) indicate that the duration of sick leave may be longer among women, potentially reflecting differences in health-seeking behaviour, job characteristics, or recovery dynamics.

As expected, age emerged as an important factor of sick leave duration. Patients aged over 51 years had the longest, and those younger than 30 years had the shortest mean sick leave. This finding is consistent with reports indicating that recovery slows with age due to reduced regenerative capacity, higher prevalence of chronic conditions, and slower functional recovery. These differences were observed in both occupational and non-occupational injuries, confirming age as a universal determinant of sick leave duration (19, 20).

Furthermore, our study revealed that head and neck injuries were more common in occupational cases, likely due to specific mechanisms such as falls or impact with equipment, whereas lower extremity injuries predominated in non-occupational cases, reflecting everyday and recreational activities. Future research should aim to further disentangle the underlying mechanisms responsible for these anatomical injury patterns by incorporating more detailed exposure data, such as specific job tasks, use of personal protective equipment, and environmental risk factors. Multi-centre studies would be particularly valuable in assessing causal relationships and temporal trends. In addition, stratified analyses by occupation type, injury severity, and circumstances of injury (e.g. work-related falls versus recreational activities) could provide a more nuanced understanding of risk profiles and inform targeted prevention strategies.

Our central finding is the longer duration of sick leave for occupational injuries within the same diagnostic categories (statistically significant for chest injuries and lower extremity injuries). This suggests that factors beyond the medical diagnosis, such as work organisation, psychosocial risks, work ability assessment procedures, and compensation systems, may substantially influence the duration of absence. Longer duration observed in occupational injuries may be attributed to specific working conditions (e.g. physically demanding tasks, machinery use) and a more complex return-to-work process, which involves assessment of fitness for specific job tasks. Future studies should incorporate variables such as industry sector, job type, and injury severity.

There are several limitations to our study. The accuracy of our data depends on medical records and administrative coding, which may introduce bias. The analysis is limited to the Banja Luka region and the one-year period. Future research should cover a longer period and incorporate occupation, cause of injury, severity, and working conditions to better understand the determinants of sick leave duration. Finally, presented estimates should be interpreted cautiously, as the cost calculations were based on average gross salaries and a simplified multiplier for indirect costs, which may not fully capture variability across sectors, individual earnings, and the full spectrum of intangible and long-term economic consequences.

## CONCLUSION

Overall, our findings confirm that occupational injuries, although less frequent than non-occupational ones, impose a greater operative and economic burden due to longer sick leave and presumably higher productivity losses and healthcare and compensation expenses. They also highlight the need for a comprehensive approach to managing occupational injuries that extends beyond prevention alone. Strengthening workplace safety measures remains essential, particularly in high-risk sectors, but should be complemented by more effective rehabilitation and return-to-work programmes aimed at reducing the duration of sickness absence. Special attention should be given to older workers, who may require tailored interventions due to prolonged recovery times.

In addition, our results suggest that compensation systems warrant careful evaluation. While ensuring adequate financial protection for injured workers is crucial, policy adjustments may help reduce potential misuse and encourage timely, medically justified return to work without creating financial pressure that could lead to premature reintegration.

Overall, integrating preventive strategies with improved post-injury management – including early rehabilitation and coordinated return-to-work pathways – has the potential to significantly reduce both the length of sick leave and the associated economic burden.
